# Editorial: Animal Welfare - Volume II: Using Bio-sensing Devices to Assess Farm Animal Welfare

**DOI:** 10.3389/fphys.2022.848955

**Published:** 2022-02-11

**Authors:** Jeroen Brijs, Andreas Fahlman, Martin Fore, Xavier Manteca

**Affiliations:** ^1^Hawai'i Institute of Marine Biology, University of Hawai'i at Manoa, Honolulu, HI, United States; ^2^Fundación Oceanografic de la Comunidad Valenciana, Valencia, Spain; ^3^Kolmården Wildlife Park, Kolmården, Sweden; ^4^Global Diving Research SL, Valencia, Spain; ^5^Department of Engineering Cybernetics, Norwegian University of Science and Technology, Trondheim, Norway; ^6^School of Veterinary Science, Universitat Autònoma de Barcelona, Barcelona, Spain

**Keywords:** animal welfare, bio-logging, sensors, precision livestock farming, fish, aquaculture, cattle

Animal welfare has become an essential element of modern animal production. First and foremost, animal welfare is grounded on ethical concerns that derive from the fact that farm animals are sentient beings (i.e., they are able to suffer and experience emotions; Le Neindre et al., 2017). Societal concern over the welfare of farm animals has increased recently and a growing number of consumers in many countries now demand that farm animals are reared, transported, and slaughtered as humanely as possible. Improving animal welfare may have additional benefits. For example, since many welfare problems can detrimentally affect production (*via* impaired appetite, growth, immune responses and reproduction), improving farm animal welfare can have positive effects on the quantity and quality of the final marketed product (Ashley, 2007). Improving animal welfare is also one of the strategies that may contribute to reducing the use of antimicrobials in farm animals (EMA and EFSA, 2017), which will likely benefit human health in the long-term.

Developing and validating science-based tools to objectively assess the welfare of farm animals is necessary to identify problems and monitor progress when strategies are being implemented to enhance and assure welfare. Assessment protocols to improve welfare in farm animals are also needed for certification schemes, which have become widespread in many countries. Although several protocols have been developed to assess the welfare of farm animals (e.g., Botreau et al., 2007), these have several limitations. For example, most current methods to assess welfare can identify existing issues but fail to anticipate future welfare problems and hence are not helpful to implement preventative measures. Also, existing protocols are typically based on focal assessments and only provide information for short periods of time. However, the welfare status of animals evolves over time as a dynamic interaction between the animal and its environment, and current assessment protocols do not enable a life-long evaluation of animal welfare. Finally, most of the existing welfare assessment tools are intended to monitor welfare at a group level and limited attention is given to individual animals.

This Research Topic includes six studies -one on cattle and five on farmed fish- that show the potential of using biosensors to overcome the limitations of existing animal welfare assessment protocols. In the study on cattle welfare, Palacios et al. use implantable bio-loggers to measure the effect of high grazing density on the circadian rhythms of temperature, heart rate, and activity. This Research Topic provides an example of how individual, continuous measurements can provide information on how animals cope with their environment, and the results suggested that a high stocking density may exacerbate the competition for valuable resources.

Three of the farmed fish studies focused on quantifying swimming activity. Arechavala-Lopez et al. placed gilthead seabream (*Sparus aurata*) in swim tunnels to investigate whether observed swimming performance and body movements could be assessed as a welfare index using transmitters measuring acceleration. While these devices did not capture all aspects of seabream activity, they showed promise as an efficient tool for detecting behavioral changes during production. Georgopoulou et al. studied European seabass (*Dicentrarchus labrax)* in a recirculation aquaculture facility. The movement was recorded using video footage and computer vision methods to extract measures of swimming speed and direction, as well as surface attraction. Their results implied that this method was suitable for detecting both speed and direction, and that these parameters could be useful in monitoring behavioral states in seabass. Stockwell et al. studied large-scale spatial movements, such as distance to the cage center and depth, movement speeds and turning angles in Atlantic salmon (*Salmo salar*) using 3D-tracking. This method seemed suitable for monitoring farmed fish, and interestingly the main indicators were susceptible to changes due to both environmental events (e.g., changes in stratification) and cage management practices (e.g., feeding). The other two Research Topics measured heart rate, providing a direct physiological focus, and were both conducted with Atlantic salmon in sea-cages. Warren-Myers et al. set out to explore the utility of heart rate as a parameter for long-term monitoring of fish welfare. Heart rate was found to be a reliable indicator in salmon, as it exhibited variations that were clearly linked to seasonal/diurnal variations and stress-induced elevations during crowding. Gamperl et al. sought to investigate how summer conditions of warm and potentially hypoxic water affected farmed salmon, and used devices that measured heart rate, depth and activity to cover both physiological and behavioral aspects of their responses. These biosensing devices proved useful for monitoring the conditions and states of farmed salmon and revealed that while the data was not significantly impacted by long exposure to high temperatures and moderate hypoxia, it seems that high temperatures in combination with biotic factors may be the most substantial climate-change related challenge in salmon aquaculture.

In conclusion, this Research Topic provides a snapshot of how bio-sensing devices are used as research tools for studying farmed animals by observing parameters such as position, acceleration, and heart rate. The articles therein also imply how devices that are commercially available today can be used for individual based welfare monitoring in animal production, potentially leading to future industrial applications.

## Author Contributions

All authors listed have made a substantial, direct, and intellectual contribution to the work and approved it for publication.

## Conflict of Interest

AF was employed by Fundación Oceanografic de la Comunidad Valenciana, Kolmården Wildlife Park, and Global Diving Research SL.

The remaining authors declare that the research was conducted in the absence of any commercial or financial relationships that could be construed as a potential conflict of interest.

## Publisher's Note

All claims expressed in this article are solely those of the authors and do not necessarily represent those of their affiliated organizations, or those of the publisher, the editors and the reviewers. Any product that may be evaluated in this article, or claim that may be made by its manufacturer, is not guaranteed or endorsed by the publisher.
